# Anthropogenic Natal Environmental Effects on Life Histories in a Wild Bird Population

**DOI:** 10.1016/j.cub.2014.01.040

**Published:** 2014-03-03

**Authors:** Samantha J. Cartwright, Malcolm A.C. Nicoll, Carl G. Jones, Vikash Tatayah, Ken Norris

**Affiliations:** 1 Centre for Agri-Environmental Research, School of Agriculture, Policy and Development, University of Reading, Reading RG6 6AR, UK; 2 Durrell Wildlife Conservation Trust, Les Augrès Manor, Trinity, Jersey JE3 5BP, Channel Islands, UK; 3 Mauritian Wildlife Foundation, Grannum Road, Vacoas, Mauritius

## Abstract

Recent work suggests that the environment experienced in early life can alter life histories in wild populations [1–5], but our understanding of the processes involved remains limited [6, 7]. Since anthropogenic environmental change is currently having a major impact on wild populations [8], this raises the possibility that life histories may be influenced by human activities that alter environmental conditions in early life. Whether this is the case and the processes involved remain unexplored in wild populations. Using 23 years of longitudinal data on the Mauritius kestrel (*Falco punctatus*), a tropical forest specialist, we found that females born in territories affected by anthropogenic habitat change shifted investment in reproduction to earlier in life at the expense of late life performance. They also had lower survival rates as young adults. This shift in life history strategy appears to be adaptive, because fitness was comparable to that of other females experiencing less anthropogenic modification in their natal environment. Our results suggest that human activities can leave a legacy on wild birds through natal environmental effects. Whether these legacies have a detrimental effect on populations will depend on life history responses and the extent to which these reduce individual fitness.

## Highlights

• Anthropogenic habitat in early life affects life history
• Reproductive scheduling and survival are modified
• Modified life history strategy compensates for poor start in life

## Teaser

Cartwright et al. show that there is a legacy of human activity on the life histories of a tropical wild bird. Individuals born in human-modified habitat have altered schedules of reproduction and survival over their lifetimes, which appear to be an adaptive, plastic response to environmental change.

## Results

There is increasing evidence that conditions in early life (the natal environment) can have a long-term impact on wild populations by modifying individual life histories (age-specific patterns in reproduction and survival) [1–5]. Studies to date have typically considered changes in various aspects of the natural environment in this context. In contrast, human-induced environmental change remains largely unexplored. This is potentially important because the majority of Earth’s ecosystems have been altered by human activities [9, 10]. This means that many organisms are exposed to human-modified habitats in early life, potentially modifying life histories through changes to the natal environment. This possibility and its consequences have yet to be explored in wild populations. Here, we examine how anthropogenic habitat change in the natal environment affects life histories in a wild population of the endemic forest-dwelling Mauritius kestrel (*Falco punctatus*). We used the area of agricultural habitat within the natal environment as a measure of the intensity of anthropogenic habitat change because the conversion of tropical forest to agriculture represents a major change in vegetation composition and structure, community dynamics, and ecosystem function [11]. The breeding success of kestrels is also reduced by exposure to agriculture [12]. To explore whether habitat change in the natal environment has persistent effects on life histories, we compared age-specific patterns of reproductive success and survival between birds experiencing a gradient in early life conditions ranging from predominantly forest (high-quality natal environment) to forest areas heavily modified by agriculture (low-quality natal environment).

### Age-Specific Reproductive Success

**Figure 1:**
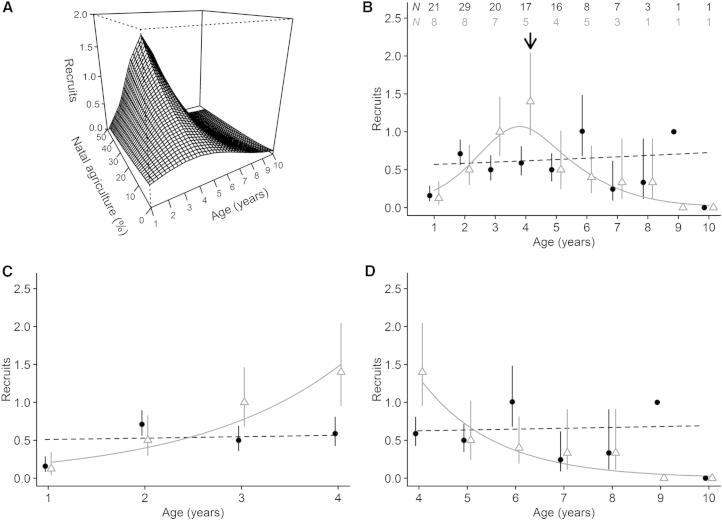
Age-Specific Patterns of Recruit Production in Female Mauritius Kestrels Filled black circles, text, and lines represent the 0% agriculture group; gray triangles, text, and lines represent the >30% agriculture group. Each data point shows the age-specific mean recruit production ± SE. Note that points are offset slightly to avoid overlap between the agriculture groups. Dashed lines indicate nonsignificant trend. Sample sizes are shown at top of (B). The surface in (A) was generated from the statistical model described in the text and shown in Table S1A. The curves in (B) were generated from the generalized additive model (GAM; 0% natal agriculture group: χ^2^_2_ = 0.703, p = 0.704; >30% group: χ^2^_2.437_ = 8.550, p = 0.040). The age of peak recruit production in the >30% agriculture group based on the GAM is shown by the arrow in (B). (C) shows age-specific recruit production prior to the peak, and (D) shows age-specific recruit production after the peak; the curves in both plots were generated from statistical models described in the text.

To compare patterns of age-specific reproductive success between birds exposed to different areas of agriculture in early life (i.e., as chicks in the nest), we used complete, spatially referenced life history data for 79 female Mauritius kestrels that fledged between 1992 and 2003 and that bred at least once (see Figure S1A available online). We used the production of recruits (i.e., number of offspring surviving to breed) as our measure of age-specific reproductive success. To separate natal from current environmental effects on recruit production, we also included individual characteristics (lifespan and age at first reproduction) and environmental effects (natal total population size, local natal population density, current breeding agriculture, and current rainfall) that might affect current reproductive success. We found that age-specific recruit production varied in response to the level of agriculture experienced in early life (age × natal agriculture interaction: χ^2^_2_ = 6.216, p = 0.045; Figure 1A; Table S1A). Birds exposed to high levels of natal agriculture had a more pronounced peak in recruit production followed by a more rapid decline later in life compared with birds exposed to low levels of agriculture. We found similar results if we used fledgling production (i.e., the number of offspring produced) as our measure of age-specific reproductive success (Figure S2A; Table S1B).

To illustrate the contrasts in life history between birds exposed to low and high levels of natal agriculture, we compared in more detail the age-specific patterns for females experiencing no natal agriculture (N = 37) and those experiencing >30% natal agriculture (N = 15). Recruit production peaked in the >30% agriculture group at 4 years of age, whereas the 0% group showed no age-specific trend (Figure 1B). In the >30% agriculture group, recruit production increased more rapidly prior to this peak (interaction between age and natal agriculture group: χ^2^_1_ = 5.422, p = 0.020; Figure 1C) and declined more rapidly after the peak (χ^2^_1_ = 5.833, p = 0.016; Figure 1D) compared with the 0% agriculture group over the same age ranges.

### Age-Specific Survival

**Figure 2:**
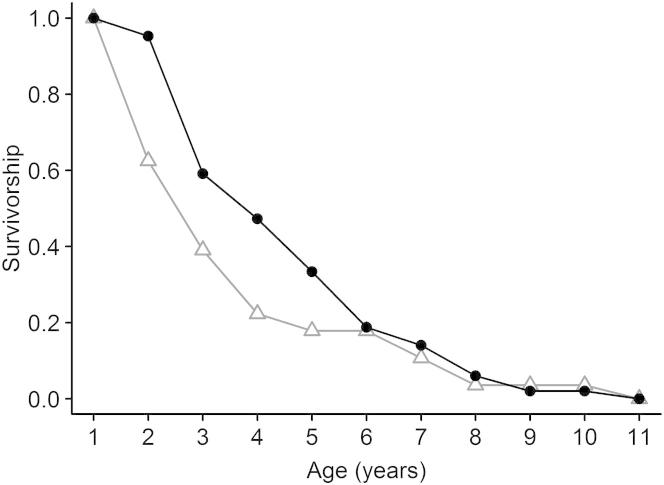
Survivorship of Females Exposed to 0% and >30% Natal Agriculture Filled black circles and line represent the 0% agriculture group; gray triangles and line represent the >30% agriculture group (N = 37 and 15 for each group, respectively). Survivorship was generated from raw data.

To investigate age-specific survival between birds exposed to differing levels of agriculture in early life, we used data for 385 female fledglings of known origin from 18 cohorts between 1992 and 2009 (Figure S1B). We found that survival rates declined as the level of natal agriculture increased, but this effect was highly age specific, occurring only in survival rates between 1 and 2 years of age (χ^2^_2_ = 8.120, p = 0.017; Table S2B). As a result, observed survivorship differed markedly between birds experiencing low (0%) and relatively high (>30%) levels of natal agriculture (Figure 2). These results might reflect a persistent effect of the natal environment on survival rates later in life. Alternatively, they may reflect differences in reproductive effort in yearling females and a cost of reproduction in terms of subsequent survival. We have shown previously in Mauritius kestrels that a reduction in reproductive effort in young females due to conservation interventions was associated with higher survival rates in subsequent years [13], so the converse is at least plausible. To explore this possibility, we compared recruit production between yearling females experiencing differing levels of natal agriculture. We distinguished females that survived to 2 years of age and those that died between 1 and 2 years of age (we have shown elsewhere that the resighting probability of females once recruited into the breeding population is ∼1 [14]). If a cost of reproduction is important, we predicted that recruit production should be higher with increasing natal agriculture, and possibly higher in nonsurvivors compared with survivors. We found a negative rather than positive relationship with natal agriculture (β ± SE [log scale] = −0.057 ± 0.037, χ^2^_1_ = 3.813, p = 0.051), no significant difference between survivors and nonsurvivors (χ^2^_1_ = 0.428, p = 0.513), and a weak interaction (survival × natal agriculture interaction: χ^2^_3_ = 6.605, p = 0.086; females = 45). In summary, we found evidence of highly age-specific survival differences in relation to anthropogenic habitat change in early life, and no evidence that this might be explained by a short-term cost of reproduction.

### Fitness Consequences

**Figure 3:**
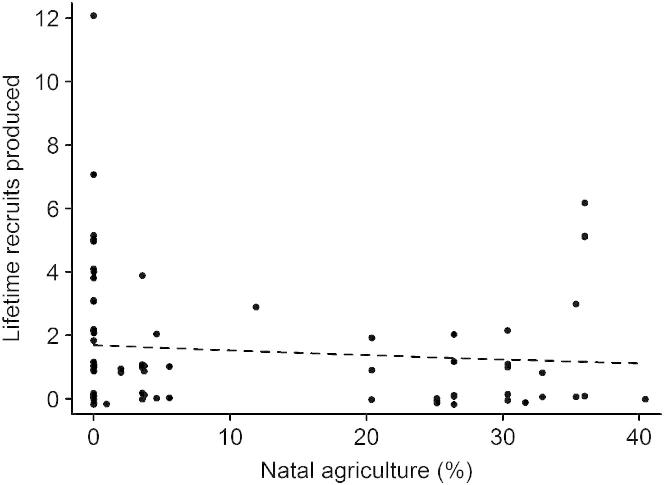
Lifetime Reproductive Success of Female Mauritius Kestrels in Relation to Natal Agriculture Data points show total recruits produced per female; the dashed line shows the nonsignificant relationship between natal agriculture and lifetime reproductive success generated from the statistical model described in the text.

Our results show that anthropogenic habitat change in early life is associated with modifications in the life histories of Mauritius kestrels. If these life history responses reduce individual fitness, then they have the potential to negatively impact population growth and viability [15]. This is a conservation issue in the case of the Mauritius kestrel, given its threatened status (classified by the IUCN as Vulnerable [16]), but it is an important general issue since many populations are likely to be exposed to anthropogenic habitat change. To explore fitness effects, we calculated the lifetime reproductive success (LRS) of each female included in the age-specific reproductive success analyses. We found no change in the LRS of females originating from territories with differing relative areas of natal agriculture (χ^2^_1_ = 2.275, p = 0.132; Figure 3). This suggests that life history responses to anthropogenic habitat change in early life are adaptive in the sense that the fitness of individuals adopting these strategies is comparable to that of individuals not exposed to human-modified habitats.

## Discussion

Our results strongly suggest that exposure to anthropogenic habitat change in early life can modify life histories in wild populations. The validity of this claim and its potential significance rest on analyses we used to describe the age-specific patterns of reproductive success and survival in the Mauritius kestrel data.

Our analysis of age-specific reproductive success in relation to natal agriculture included additional variables that potentially described the effects of the natal and current environment and the selective appearance and loss of individuals on the production of recruits (Table S1A). Furthermore, we used an analytical framework comparable to a number of other published studies [1–5] to distinguish anthropogenic natal environmental effects from these other factors. In addition, we used generalized additive models to compare patterns between birds experiencing low (0%) and high (>30%) levels of agriculture (Figure 1B), which showed that birds exposed to high levels of agriculture had a more pronounced peak in recruit production in early life. Further analysis showed that this was due to a more rapid increase in recruit production prior to this peak (Figure 1C), followed by a more rapid decline after the peak (Figure 1D). We obtained comparable results if we used fledgling rather than recruit production as our measure of reproductive success (Figure S2A; Table S1B). We therefore conclude that our results are not dependent on the measure of reproductive success we use and are unlikely to be confounded by other important ecological processes that we know affect reproductive success.

Exposure to agricultural habitats in early life also appears to affect survival, but in a highly age-dependent manner (Table S2B). Our survival analysis included a candidate model that distinguished no natal agriculture effect (model A7, Table S2A), but this received substantially less support than the most parsimonious model that distinguished a natal agriculture effect in a single age class (1- to 2-year-old birds) (model A6, Table S2A). We found no evidence that the effect on survival could be explained as a short-term response to differences in reproductive effort among yearling breeders. We have previously documented short-term increases in survival following reductions in reproductive effort due to conservation management in Mauritius kestrels [13]. If an analogous process explains the survival differences we found in relation to agriculture, then we would expect to see a positive correlation between recruit production and natal agriculture among yearling females, but we actually found the opposite trend. We therefore tentatively conclude that exposure to anthropogenic habitat change in early life might have persistent effects on survival as well as on reproductive success in Mauritius kestrels.

How might the patterns we report arise? The clear structural dichotomy between forest and agricultural habitats is likely to affect both the hunting success and prey available to breeding Mauritius kestrels. These birds are adapted to hunting in the tropical forest canopy [17], but agricultural areas contain almost no features that would provide equivalent perches for hunting or a complex canopy for concealment. Furthermore, although Mauritius kestrels are known to consume a wide range of prey including native arboreal geckos and birds; exotic lizards, birds, and small mammals; and various insects, observations of offspring provisioning at nest sites indicated that >80% of all prey items were arboreal *Phelsuma* geckos [18]. Although we lack quantitative data on habitat-specific prey availability, it is probable that *Phelsuma* geckos, which are characteristic of forest stands [19], are less abundant in agricultural areas, and previous work has shown that birds occupying agricultural territories are less likely to deliver geckos to the nest [20]. If food delivery to the nest is affected in this way, then chicks in agricultural territories could be exposed to nutritional stress at a critical time in their growth and development. Nutritional stress during early development has been shown to have detrimental long-term consequences in a range of taxa [21]. In addition, since the natal environment can indicate to developing offspring the environmental conditions they are likely to experience after maturity [7, 22], nutritional stress during early life may indicate a harsh and/or unpredictable adult environment, i.e., one in which the risk of death is great and residual reproductive value is correspondingly low [23, 24]. In these circumstances, increasing reproductive effort as a young breeder at the expense of reproductive effort or survival later in life is likely to be beneficial for fitness, which in broad terms is the life history response we observed.

Interestingly, our analysis suggests that the life history responses to anthropogenic habitat in early life we observed are adaptive in the sense that the fitness of individuals adopting these strategies was comparable to that of individuals not exposed to human-modified habitats (Figure 3). In contrast, previous studies have often shown that individuals originating in “good-quality” environments have higher fitness than individuals from “poor-quality” environments [25, 26], the so-called “silver spoon” effect [27, 28]. This effect is not universal, however. Evidence from laboratory studies suggests that individuals can fully compensate for a poor start in life by modifying their life history strategies [29]. This issue is important because if life history responses to natal environments result in reduced fitness, they can have population dynamic consequences [15]. This could be highly relevant in a conservation context because we know that habitat modification detrimentally affects many habitat specialists [30], particularly in the tropics, where the rate of forest clearance for agriculture is most rapid [31]. As a result, there is a pressing need to understand the processes and mechanisms responsible for population declines [32, 33], which must include assessing the extent to which organisms are able to adapt to and persist in human-modified environments [7]. In this wider context, we view the life history responses that we observed as a potentially adaptive plastic response to an anthropogenic environmental stress early in life. Whether or not fitness is compromised in such cases is likely to depend on the limits of phenotypic plasticity, which are currently poorly understood [34–36]. Our data suggest that Mauritius kestrels have yet to reach these limits in terms of the habitat change they are exposed to in early life, and hence the life history modifications we report have no major local conservation implications.

Taken together, our results suggest that human activities can have a persistent effect on the life histories of wild organisms through natal environmental effects. Given the ubiquity of human-induced habitat change [9, 10, 37], the patterns we report could be widespread but remain poorly documented due to the short-term nature of most studies that attempt to quantify only the immediate impact of habitat change on fitness traits, e.g. [38]. This approach ignores the fact that changes to reproductive output and survival may be delayed in time and observed in different habitats from those ultimately driving the changes as a result of natal dispersal. The extent to which this matters in terms of assessing the impact of anthropogenic habitat change on wild populations depends on the relative importance of delayed life history effects and the current environment. This issue can only be addressed using longitudinal data, highlighting the importance of long-term population studies in understanding the ecology of habitat change.

## Supplemental Information

Document S1. Figures S1 and S2, Tables S1 and S2, and Supplemental Experimental Procedures
