# Supplementary material for: Anthropogenic Natal Environmental Effects on Life Histories in a Wild Bird Population
Source: Curr Biol. 2014 Mar 3;24(5):536–40. doi: 10.1016/j.cub.2014.01.040 (PMC3969248; doi:10.1016/j.cub.2014.01.040)
Supplement: Document S1. Figures S1 and S2, Tables S1 and S2, and Supplemental Experimental Procedures [file mmc1.pdf]

Current Biology, Volume *24*

Supplemental Information

Anthropogenic Natal Environmental Effects on Life Histories in a Wild Bird Population

Samantha J. Cartwright, Malcolm A.C. Nicoll, Carl G. Jones, Vikash Tatayah, and Ken Norris

A

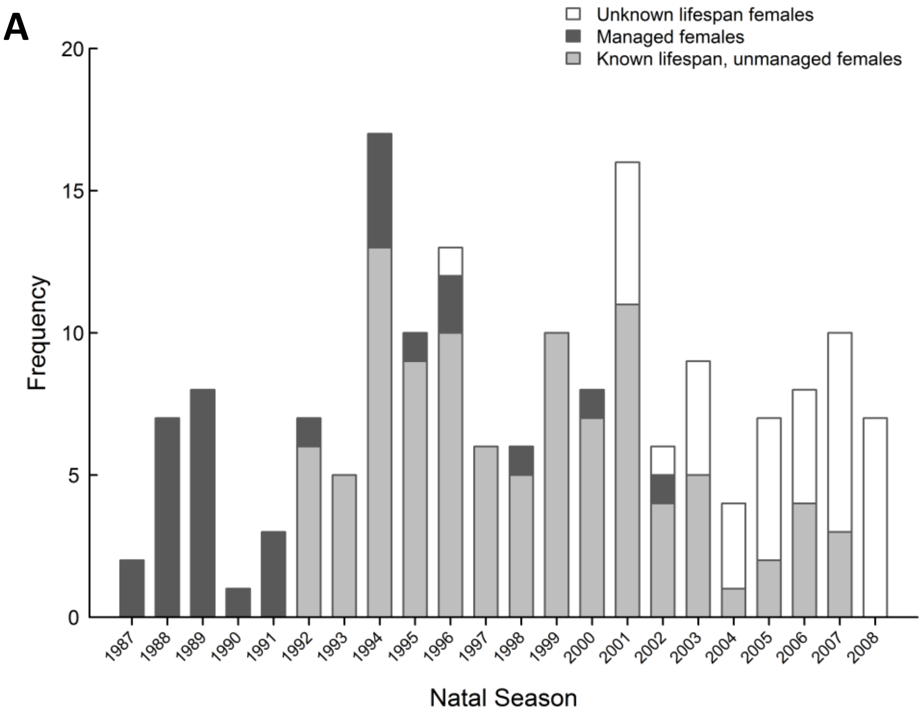

B

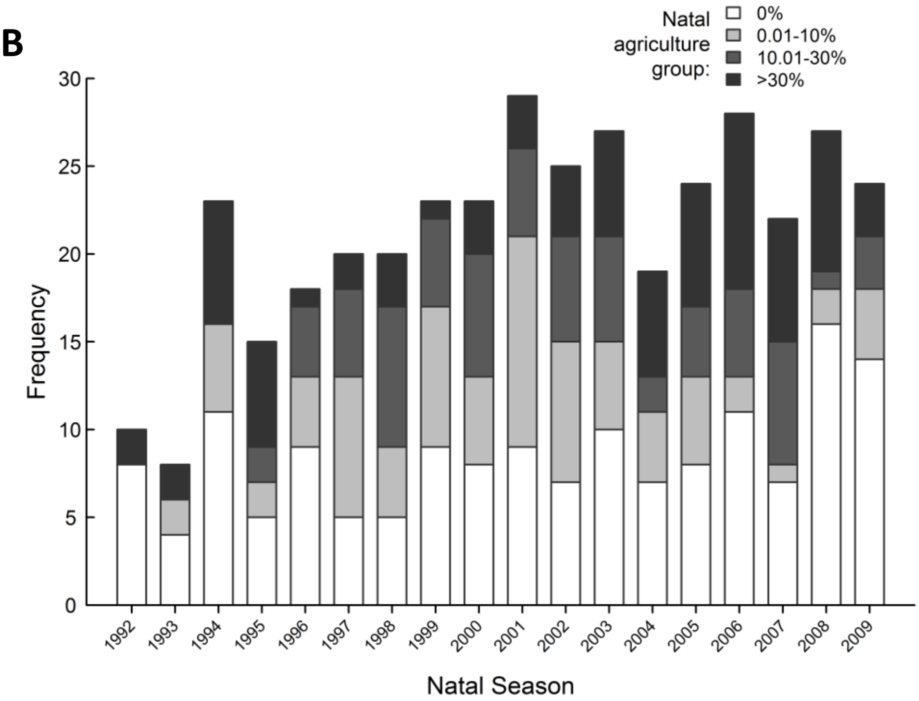

Figure S1. (A-B) Samples of females used in life history analyses, related to Figure 1, Figure 2 and Supplemental Experimental Procedures

Plot (A) shows natal cohorts of breeding female Mauritius kestrels since reintroduction to the Bambous mountains study area (N=170). All females included in plot bred at least once (i.e. laid a clutch). Light grey component (known lifespan, unmanaged females) available for analyses of reproductive scheduling (N=101). The sample for analysis of recruit production is limited to 79 females with their final breeding attempt in, or prior to, the 2005 season. Plot (B) shows cohorts of wild-hatched females included in the CMR survival analysis (N=385). Re-sighting data was available for the 2010 season. Females from cohorts prior to 1992 were excluded due to conservation management (e.g. supplemental feeding) carried out that might bias estimates of survival. Female fledglings specifically excluded were those from the site "Domaine du Chasseur Car Park" (due to on-going supplemental feeding) and a female fledging from "Domaine du Chasseur 8" during the 2008 season (also due to feeding).

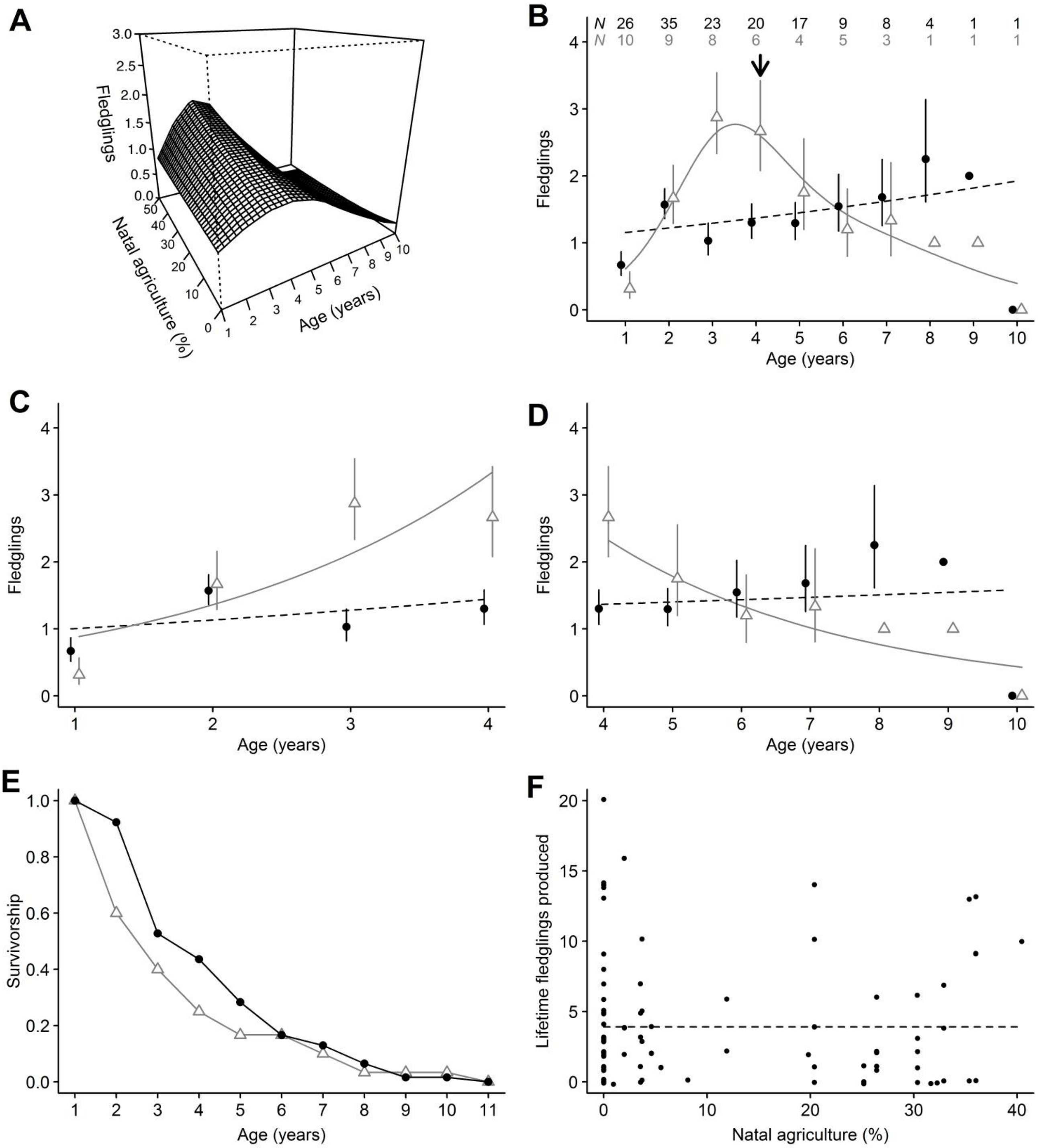

Figure S2. (A-F) Age-specific patterns of fledgling production, survivorship and lifetime reproductive success in female Mauritius kestrels, related to Figure 1 and Supplemental Experimental Procedures

Surface in (A) was generated from the statistical model described in the text and shown in Table S1B. In (B-D) filled black circles, text and lines represent the 0% agriculture group; grey triangles, text and lines represent the >30% agriculture group. Points show the age-specific mean fledgling production ± SE. Points are offset slightly to avoid overlap between the agriculture groups. Dashed lines indicate non-significant trend. Sample sizes are shown in (B). Curves in (B) were generated from the GAM (0% natal agriculture group: $\chi^2_1 = 2.809$, P = 0.094; >30% group: $\chi^2_{4.342} = 23.952$, P <0.001). The age of peak fledgling production in the >30% agriculture group based on the GAM is shown by an arrow. Plot (C) shows age-specific fledgling production by each group of birds prior to the peak (interaction between natal agriculture group and age: $\chi^2_1 = 7.084$, P = 0.008) and (D)

shows it after the peak (interaction between natal agriculture group and age: χ^2_1 = 5.758, P = 0.016), with the curves in both plots generated from statistical models described in the text. In (E) filled black circles represent the observed survivorship of the 0% agriculture birds (N=46); grey triangles represent the >30% agriculture birds (N=17). In (F) points are the observed data for total fledglings produced per female; line shows non-significant relationship (χ^2_1 <0.001, P = 0.996) generated from statistical model described in the text.

Table S1 (A-C). Model of age-specific reproductive output, related to Figure 1 and Supplemental Experimental Procedures

(A) - recruits

Parameter	Estimate	SE	z	P
Intercept	-0.416	0.980	-0.425	0.671
December rainfall (wet)	-0.290	0.191	-1.521	0.128
**Breeding agriculture (%)**	**-0.023**	**0.008**	**-2.866**	**0.004**
**Natal Density**	**-0.044**	**0.014**	**-3.189**	**0.001**
Natal Local Density	0.077	0.085	0.911	0.362
AFR (2+ years)	1.109	1.207	0.919	0.358
Age	0.797	0.661	1.206	0.228
Age^2	-0.101	0.102	-0.981	0.327
Lifespan	0.157	0.137	1.148	0.251
Natal agriculture (%)	-0.045	0.035	-1.296	0.195
Age x Lifespan	-0.084	0.082	-1.021	0.307
Age^2 x Lifespan	0.010	0.011	0.890	0.374
AFR (2+ years) x Age	-0.628	0.641	-0.980	0.327
AFR (2+ years) x Age^2	0.080	0.076	1.050	0.294
**Age x Natal agriculture (%)**	**0.041**	**0.019**	**2.106**	**0.035**
**Age^2 x Natal agriculture (%)**	**-0.005**	**0.002**	**-2.134**	**0.033**
Lifespan x Natal agriculture (%)	-0.002	0.003	-0.714	0.475
AFR (2+ years) x Natal agriculture (%)	-0.004	0.015	-0.264	0.792

(B) - fledglings

Parameter	Estimate	SE	z	P
Intercept	0.591	0.451	1.309	0.190
December rainfall (wet)	-0.136	0.105	-1.293	0.196
Breeding agriculture (%)	-0.007	0.004	-1.716	0.086
**Natal Density**	**-0.036**	**0.007**	**-4.986**	**<0.001**
**Natal Local Density**	**0.145**	**0.048**	**3.013**	**0.003**
AFR (2+ years)	-0.415	0.530	-0.784	0.433
Age	0.454	0.256	1.774	0.076
**Age^2**	**-0.073**	**0.036**	**-2.018**	**0.044**
Lifespan	0.016	0.062	0.259	0.796
Natal agriculture (%)	-0.002	0.015	-0.161	0.872
Age x Lifespan	-0.019	0.029	-0.635	0.525
Age^2 x Lifespan	0.005	0.004	1.484	0.138
AFR (2+ years) x Age	0.207	0.236	0.879	0.380
AFR (2+ years) x Age^2	-0.023	0.024	-0.973	0.330
Age x Natal agriculture (%)	0.012	0.008	1.583	0.113
**Age^2 x Natal agriculture (%)**	**-0.002**	**0.001**	**-1.969**	**0.049**
Lifespan x Natal agriculture (%)	-0.001	0.002	-0.618	0.537
AFR (2+ years) x Natal agriculture (%)	0.003	0.008	0.326	0.745

(C) – clutch size

Parameter	Estimate	SE	z	P
Intercept	1.098	0.304	3.616	<0.001
December rainfall (wet)	0.046	0.072	0.645	0.519
Breeding agriculture (%)	<0.001	0.003	-0.020	0.984
Natal Density	-0.009	0.005	-1.736	0.083
Natal Local Density	0.033	0.032	1.030	0.303
AFR (2+ years)	-0.272	0.350	-0.778	0.436
Age	0.132	0.163	0.809	0.419
Age^2	-0.025	0.023	-1.084	0.278
Lifespan	0.009	0.038	0.236	0.813
Natal agriculture (%)	0.009	0.009	0.958	0.338
Age x Lifespan	<0.001	0.018	-0.025	0.980
Age^2 x Lifespan	0.001	0.002	0.581	0.561
AFR (2+ years) x Age	0.108	0.158	0.683	0.494
AFR (2+ years) x Age^2	-0.012	0.016	-0.783	0.433
Age x Natal agriculture (%)	-0.001	0.005	-0.274	0.784
Age^2 x Natal agriculture (%)	<0.001	<0.001	0.351	0.726
Lifespan x Natal agriculture (%)	-0.001	0.001	-0.606	0.544
AFR (2+ years) x Natal agriculture (%)	-0.002	0.007	-0.274	0.784

Table (A) summarizes the results of the global generalised linear mixed model (GLMM) of age-specific recruit production by female Mauritius kestrels. Table (B) summarizes the results of GLMM of age-specific fledgling production. Table (C) summarizes the results of the GLMM of age-specific clutch size. All three models were fit assuming Poisson errors, a log link function and with female identity as a random effect (variance component ± std. dev. for recruits: <0.001 ± <0.001; fledglings: 0.003 ± 0.051; clutch size: <0.001 ± <0.001). Significant (P <0.05) terms are shown in bold.

Table S2. (A-B) Candidate and final survival models for female Mauritius kestrels, related to Figure 2 and Supplemental Experimental Procedures

(A)

ID	Recapture probability	Survival probability	np	Dev	AICc	ΔAICc	*w*
R1	1,2,3,4,5+	1_{t},2,3,4,5+	27	790.505	1616.623	0.000	0.466
R2	1,2,3,4+	1_{t},2,3,4,5+	26	797.867	1621.855	5.232	0.034
R3	1,2,3+	1_{t},2,3,4,5+	25	810.518	1632.380	15.758	<0.001
R4	1,2+	1_{t},2,3,4,5+	24	821.234	1640.977	24.354	<0.001
J1	1,2,3,4,5+	1_{t},2,3,4,5+	27	790.505	1616.623	22.512	<0.001
J2	1,2,3,4,5+	1_{den35},2,3,4,5+	11	801.479	1594.111	0.000	0.984
J3	1,2,3,4,5+	1_{den25},2,3,4,5+	11	811.427	1604.058	9.948	0.007
J4	1,2,3,4,5+	1*N,2,3,4,5+	11	810.857	1603.488	9.377	0.009
A1	1,2,3,4,5+	$1_{den35*NatAg}$,2_{NatAg},3_{NatAg},4_{NatAg},$5+_{NatAg}$	16	1562.169	1594.806	4.698	0.047
A2	1,2,3,4,5+	$1_{den35*NatAg}$,2_{NatAg},3_{NatAg},4_{NatAg},5+	16	1563.169	1595.806	5.698	0.029
A3	1,2,3,4,5+	$1_{den35*NatAg}$,2_{NatAg},3_{NatAg},4,5+	15	1563.302	1593.864	3.756	0.075
A4	1,2,3,4,5+	$1_{den35*NatAg}$,2_{NatAg},3,4,5+	14	1563.605	1592.095	1.988	0.183
A5	1,2,3,4,5+	$1_{den35*NatAg}$,2,3,4,5+	13	1566.746	1593.170	3.063	0.107
A6	1,2,3,4,5+	1_{den35},2_{NatAg},3,4,5+	13	1563.683	1590.108	0.000	0.493
A7	1,2,3,4,5+	1_{den35},2,3,4,5+	11	1571.803	1594.111	4.003	0.067

(B)

Survival			*Recapture*		
Parameter	Estimate	SE	Parameter	Estimate	SE
Intercept	0.911	0.151	Intercept	3.516	0.583
Natal agriculture (%)	-0.005	0.007	Age 1	-3.221	0.608
Age 1	-1.164	0.218	Age 2	-2.520	0.622
Age 2	1.193	0.509	Age 3	-2.323	0.639
Age 3	0.539	0.344	Age 4	-1.738	0.691
Age 4	0.703	0.406			
Age 1 x den<35	1.381	0.346			
Age 2 x Natal agriculture (%)	-0.036	0.020			

In (A), models R1-4 are candidate models of recapture; J1-4 are candidate models of juvenile female survival and A1-7 are candidate models of female survival. Numbers 1-5+ indicate female age classes and + indicates that all subsequent ages are included in a class; t indicates time dependence for the age class; den35 / den25 indicate the threshold population density that divides juvenile survival rates into two classes; N indicates population density as a linear constraint; NatAg indicates natal agriculture as an individual covariate. Presented, are number of parameters (np), deviance, Akaike's Information Criterion corrected for small sample sizes (AICc,[S1]), difference in AICc from the best model (ΔAICc), and Akaike's weights (*w*). For models J1-J4 and A1-7, recapture was modelled as time constant with the same age classes used for survival. The most parsimonious model from the candidate set A1-7 was model A6. Table (B) summarizes the results of model A6. Probability estimates are in logits and age class 5+ is represented by values for the intercept.

Supplemental Experimental Procedures

Study Population and Area

The Mauritius kestrel is a small forest-dwelling falcon endemic to Mauritius that is primarily adapted to hunting arboreal geckos of the *Pheluma* genus [S2,S3]. Formerly Critically Endangered, it suffered a population decline that corresponded with human colonisation of the island and widespread habitat conversion for agriculture, followed by a rapid 20th Century decline as a consequence of introduced predators, pesticides and human persecution [S4]. The species reached a low of 4 wild individuals in 1974 [S2], but was the focus of an intensive conservation programme involving captive breeding and reintroduction to areas from which it had previously been extirpated and to bolster the remaining wild population [S5].

Our study population is one of two remaining wild, distinct populations of the species. This population was extirpated from the Bambous mountains in southeast Mauritius (20.3°S, 57.7°E) by the 1960s before being reintroduced in 1987. The Bambous mountains vary in elevation from sea level to 626m and consist of a single 15km spine running East-West and a number of southward-running spurs. The study area is predominantly forested, surrounded by a wide buffer of open agricultural land (predominantly sugar cane). The non-agricultural habitat comprises a heterogeneous matrix in which native forest is invaded to varying degrees by exotic tree and plant species such as Travellers palm *Ravenala madagascariensis*, strawberry guava *Psidium cattleianum* and cultivated eucalyptus *Eucalyptus globulus* and is divided by patches of grassland managed for introduced Java deer *Cervus timorensis*.

The Mauritius kestrel has a monogamous, territory-based breeding system. Breeding pairs defend an area immediately around the nest of approximately 1km^2 [S5,S6]. Nests are located in nest boxes or natural cavities in rock faces and trees (ratio 6:3:1) and the population is monitored intensively every breeding season. The breeding season spans the Southern Hemisphere summer (but is referred to by the first of the two calendar years it spans). The earliest eggs are laid at the beginning of September (clutch size 2-4), the majority of chicks are fledged by the end of December and the latest fledglings leave the nest by early February. Kestrels are single brooded but can lay a second clutch if the first fails or once offspring have fledged, although in this latter case the second clutch has never been known to produce fledglings. Juveniles are independent at three months and can breed in their first year. Female Mauritius kestrels can begin breeding as late as 6 years of age, but the majority of the study females (83%) had recruited before 3 years, with two thirds of these (68%) recruiting at one year of age. Once established as a breeder, females typically breed every year. Between 1987 and 1989 captive-produced birds were released into the Bambous mountains and until 1994 some, or all, breeding attempts were managed as part of the recovery programme [S7]. Since the population's inception, almost all individuals have been individually marked and all breeding attempts intensively monitored [S7]. Every breeding season all breeders are identified by their unique colour ring combinations and repeated visits to nests establish the timing of laying, clutch size, brood size, and number of fledged young. Chicks are ringed and sexed in the nest based on biometrics at 12-28 days old [S7,S8] such that almost all individuals are uniquely marked with less than 10% of new breeders un-ringed by 1995 [S9]. If kestrels enter the breeding population as un-ringed individuals due to fledging from the few nests inaccessible for ringing (<6 by 1996), they are trapped so that their subsequent life history is recorded but their origin remains unknown. The population appears to have reached carrying capacity [S10], numbering 49 pairs in the 2009 season and spanning an area of approximately 163km^2. The study area is bounded by agricultural land, which along with the relatively short dispersal distances of kestrels [S5,S11] means that this is a closed population.

Data

Breeding and re-sighting data was available for more than 1000 individuals in our study population from 1987 until 2010. However, our analysis of age-specific reproductive output (in terms of fledglings and clutch size) was restricted to the 101 unmanaged females fledged from cohorts in the period 1992 to 2007 with complete, spatially-referenced life history information (i.e. of known origin, breeding and lifespan, Figure S1A). The population has experienced conservation management as part of the reintroduction but no managed females were included in our analyses to avoid potential life history bias as a result of nest manipulation [S8]. The most recent breeding records for this sample population were in 2008, thereby allowing a two-year re-sighting window which is sufficient to confirm the final breeding attempt as the age of last breeding (unpublished data). As Mauritius kestrels generally recruit up to 5 years of age, our analysis of age-specific production of recruits utilised only the females with complete life history data up to and including the 2005 season (N = 79 females) to allow a sufficient re-sighting window for the recruitment of fledglings produced. During monitoring, each nest is visited at least once during the laying period and again once the all eggs have been laid in order to establish full clutch size. A pre-fledging nest visit when the young are ~29 days old indicates the number expected to fledge and this value is used as the number of fledglings produced by a breeding attempt. Recruits are defined as marked fledglings that have subsequently been recorded breeding. Only the breeding attempt producing fledglings in each breeding season was used in analyses, but if more than one clutch was laid and neither resulted in fledglings then data relating to the first clutch was used (e.g. clutch size, breeding territory and associated area of agriculture). Our survival analyses used data for 385 unmanaged female fledglings from 18 fledged cohorts in the period 1992 to 2009 and included re-sighting data up to and including 2010 (Figure S1B).

The location of nest boxes and cavities has not changed over time and there has been no appreciable change in habitat composition for the duration of the study, enabling us to characterise nest territories according to the proportion of different habitat types they contain. Agriculture was one of seven habitat classifications on the digital habitat map, with the others comprising 5 forest types (native, semi-invaded, invaded and plantation forest and *Ravenala madagascariensis*), plus grassland [S11]. For the purposes of this study only the area of agricultural habitat occurring within a 1km^2 area surrounding the nest site was used in analyses. Daily rainfall data was available for the duration of the study [S12].

Statistical Models

Age-specific reproductive output

We explored whether natal agriculture affected an individual's age-specific reproductive output by examining three reproductive traits: clutch size, number of fledglings and number of recruits. We focussed on recruits produced in the main text because this trait is most closely associated with fitness, but present the results for fledglings and clutch size in Tables S1B and S1C and the age-specific patterns of fledgling production, survival and lifetime reproductive success in Figure S2.

In order to model age-specific reproductive output over the entire lifespan we used generalised linear mixed-effects models (GLMMs) in the statistical program R [S13] with the lme4 library [S14], which allowed us to incorporate female identity as a random effect as recommended by [S15] in order to account for repeated (i.e. non-independent) observations from the same female. We used Poisson errors and a log link function for recruit production, fledgling production and clutch size. For each measure of reproductive success, the model included the same set of variables intended to describe the natal and adult environment. We included the relative area of agriculture within the natal and breeding territories. To test for the degree of correlation between the two, we performed a linear regression of the arcsine-transformed mean proportion of agriculture in adult breeding territories with the proportion of natal agriculture as the sole predictor. The two were

significantly positively correlated (F_1 = 23.4; P <0.001), however natal agriculture explained less than 20% of the variation in mean breeding agriculture (R^2 = 0.183). As a result, it is possible to statistically separate the effects of current and natal agriculture on reproductive success. Given the increase in population size prior to and during the study period [S16], we also tested whether there was any significant relationship between the total population size and the mean area of agriculture in occupied territories during the study period. There was no significant increase in the proportion of agriculture within breeding territories over time (1992-2007; F_1 = 1.841; P = 0.178), suggesting that any effect of agriculture is unlikely to be confounded by changes in density over time. Furthermore, total population size and local population density were included in our GLMMs (see below).

In order to see whether natal agriculture affected an individual's age-specific reproductive output, we constructed a global model containing age and natal agriculture as the fixed effects of interest. We also included an interaction between natal agriculture and both age and it's square to determine whether age-specific reproductive output differed in relation to natal agriculture. To further characterise the natal environment we included the total population size (i.e. number of breeding pairs) during the natal season as this is negatively associated with juvenile survival [S17] and may thus affect the life histories of surviving individuals. For the same reason we also included the local population density (i.e. the number of breeding pairs within 1km^2 of the natal site) during the natal season. To further characterise the breeding environment, we included current breeding agriculture as a linear covariate and a two-level factor indicating whether the current year of breeding had a wetter or drier than average December (mean ± SE from 1993 to 2008 = 241.89mm ± 44.52), as rainfall in the month of December has been associated with reduced nestling survival [S18] and breeding success [S19]. Finally, to account for the selective appearance or disappearance of phenotypes with age that could result in contrasting life histories [S15,S20-23], we included age at first reproduction (AFR; median = 1, range = 1:6) as a two-level factor (AFR=1; AFR>1) and lifespan (median = 4, range = 1:12) as a covariate [S15,S22] and first-order interactions between each of lifespan and AFR with age/age^2. Lifespan was defined as the age at last breeding once females were not sighted for two consecutive years and were therefore assumed to have died. We also included interactions between each of lifespan and AFR with natal agriculture to control for any potential bias arising if the component of short-lived females from recent cohorts were more likely to originate from agricultural territories and have distinct life histories. The parameter estimates from the models for each trait are presented in Tables S1A-C.

For each measure of reproductive success, there was no compelling evidence of over-dispersion in the model (variance/mean ratio: recruits = 1.127; fledglings = 1.105; clutch size = 0.381), so the interaction between natal agriculture and age/age^2 was tested for significance using likelihood ratio tests (LRT; chi-squared test statistic). If this interaction was significant it would indicate that individual-level patterns of age-specific reproduction vary depending on the amount of agriculture experienced in early life.

For the subsequent analyses, only the age-specific recruit and fledgling production were examined. For each measure of reproductive success we took a subset of females exposed to either 0% or >30% natal agriculture to contrast the life histories of birds experiencing the lowest and highest natal agriculture conditions we observed. Using these two groups, we modelled age-specific reproduction as a function of age-by-natal agriculture group using generalised additive models (GAMs) in the mgcv library [S24] in order to identify the age and measure of peak reproductive output in each group. The shape of the GAM for recruits is presented in Figure 1B and that for fledglings is shown in Figure S2B. Having identified the age of peak reproductive output in each natal agriculture group using GAMs (>30% agriculture peak = 4 years for both recruit and fledgling production), we split the data into pre- and post-peak phases. This allowed us to separately compare changes in reproductive output with age between the natal agriculture groups during these two phases. To do this, we fit GLMMs with female identity as the random effect and an age × natal agriculture group interaction (without the quadratic age term) for the pre- and post-peak phases and tested the significance of the interaction with LRT. For the pre-peak phase, a significant interaction would indicate that the rate of increase differed between 0%

and >30% natal agriculture. For the post-peak analysis, a significant interaction between age and natal agriculture group would indicate variation in the rate of senescence between 0% and >30% natal agriculture birds [S21,S25].

Age-specific survival

We used a capture-mark-recapture framework in program MARK [S26] to account for imperfect recapture probability of fledglings in estimates of survival. Initially, we assessed what parameter structure would best describe recapture and juvenile survival. Focussing first on recapture, we used a five age-class structure (juvenile, 2, 3, 4, and ≥5 years of age) in both survival and recapture based on previous work [S8] and time-dependence in juvenile survival also based on previous work [S7,S17] as our umbrella model (model R1, Table S2A). This umbrella model fit the data adequately with no detectable over-dispersion (P = 0.44; bootstrapped goodness-of-fit ĉ 1.007; median ĉ 0.974; Cooch and White [S27]). Candidate models had progressively fewer age classes in recapture but the most parsimonious model was the umbrella model. Our subsequent modelling of juvenile survival structure proceeded using this recapture age structure. Juvenile survival has previously been shown to be time-dependent with a threshold non-linear response to population density (i.e. density-independent survival below a population density of 25 pairs and a density-dependent linear decline in survival above 25 pairs) best describing the variation when both sexes are modelled [S7,S17]. Using the same five age-class structure for both survival and recapture probability, with time-dependent juvenile survival as our umbrella model (model J1, Table S2A), we then tested different structures of juvenile survival to determine which would best describe it. Visual inspection of the estimates from the umbrella model suggested that juvenile survival declined following a threshold population density of 35 pairs and therefore a two-level grouping factor of ≤35 and >35 breeding pairs (reached during the 1996 breeding season) might best describe the juvenile survival response. We then compared the fit of a model containing this density structure (model J2) with a model in which the threshold was 25 pairs (model J3). We also tested the effect of a linear density constraint describing juvenile survival (model J4). All models in the juvenile survival analysis used a logit link function. Of our candidate models, the one best describing the variation in juvenile female survival was model J2, so density dependence in this form was applied to juvenile survival in all subsequent models.

Our umbrella model for the main survival analysis therefore comprised five age classes in survival (juvenile, 2, 3, 4 and 5+ years of age), density dependence in juvenile survival and the same age-class structure in recapture probability (model A1, Table S2A). To each of the survival age classes we applied a natal agriculture individual covariate. Our candidate models were nested within the umbrella model such that each model was a simplified version, achieved by sequentially dropping the natal agriculture effect from each age class. The resulting set of candidate models is shown in Table S2A (models A1-7). We used AICc (Akaike's Information Criterion corrected for small sample size [S1]) to identify the most parsimonious model (i.e. ΔAICc≥2) and LRT to present the significance of the natal agriculture effect. If natal agriculture remained a term in the most parsimonious model, this implied that female survival varied with natal agriculture. The estimates from the most parsimonious model are shown in Table S2B.

Fitness consequences

In order to understand the consequences of the life-history strategies associated with varying levels of natal agriculture on fitness, we calculated the lifetime reproductive success (LRS) for females included in the study, in the form of total recruits produced (Figure 3) and total fledglings produced (Figure S2F). We then tested whether the LRS varied in relation to natal agriculture using a simple GLM with Poisson errors and a log link. The natal agriculture term was tested using LRT and if there was no statistically significant effect on LRS, it would suggest that there was no variation in fitness with the level of natal agriculture experienced. Therefore females with a life history pattern consistent with higher agriculture natal territories would achieve the same

fitness as females with a life history pattern associated with lower agriculture natal territories. If this is the case then the change in life history patterns can be said to be adaptive.

Supplemental References

S1. Burnham, K.P., and Anderson, D.R. (2002). Model Selection and Multimodel Inference: A Practical Information-Theoretic Approach, 2nd Edition, (New York: Springer).

S2. Jones, C.G. (1987). The larger land birds of Mauritius. In Studies of Mascarene Island Birds, A.W. Diamond, ed. (Cambridge: Cambridge University Press), pp. 208-300.

S3. Groombridge, J.J., Nicoll, M.A., Jones, C.G., and Watson, J. (2004). Associations of evolutionary and ecological distinctiveness amongst Indian Ocean kestrels. In Raptors Worldwide, Proceedings of the VI World Conference on Birds of Prey and Owls, R.D. Chancellor and B.-U. Meyburg, eds. (Berlin: World Working Group on Birds of Prey and Owls), pp. 679-692.

S4. Jones, C.G., and Owadally, A.W. (1985). The status ecology and conservation of the Mauritius kestrel. In Conservation Studies on Raptors, I. Newton and R.D. Chancellor, eds. (Cambridge: ICBP), pp. 211-222.

S5. Jones, C.G., Heck, W., Lewis, R.E., Mungroo, Y., Slade, G., and Cade, T. (1995). The restoration of the Mauritius kestrel *Falco punctatus* population. Ibis *137*, S173-S180.

S6. Carter, J.E., and Jones, M.H. (1999). Habitat composition of Mauritius kestrel home ranges. J Field Ornithol. *70*, 230-235.

S7. Nicoll, M.A.C., Jones, C.G., and Norris, K. (2003). Declining survival rates in a reintroduced population of the Mauritius kestrel: evidence for non-linear density dependence and environmental stochasticity. J Anim. Ecol. *72*, 917-926.

S8. Nicoll, M.A.C., Jones, C.G., and Norris, K. (2006). The impact of harvesting on a formerly endangered tropical bird: insights from life-history theory. J Appl. Ecol. *43*, 567-575.

S9. Groombridge, J.J., Bruford, M.W., Jones, C.G., and Nichols, R.A. (2001). Evaluating the severity of the population bottleneck in the Mauritius kestrel *Falco punctatus* from ringing records using MCMC estimation. J Anim. Ecol. *70*, 401-409.

S10. Butler, S.J., Benton, T.G., Nicoll, M.A.C., Jones, C.G., and Norris, K. (2009). Indirect population dynamic benefits of altered life-history trade-offs in response to egg harvesting. Am. Nat. *174*, 111-121.

S11. Burgess, M.D., Black, R.A., Nicoll, M.A.C., Jones, C.G., and Norris, K. (2009). The use of agricultural, open and forest habitats by juvenile Mauritius kestrels (*Falco punctatus*). Ibis *151*, 63-76.

S12. Senapathi, D., Underwood, F.M., Black, E.C., Nicoll, M.A.C., and Norris, K. (2010). Evidence for long-term regional changes in precipitation on the East Coast mountains in Mauritius. Int. J Climatol. *30*, 1164-1177.

S13. R Core Team (2013). R: A language and environment for statistical computing, version 3.0.1. (Vienna, Austria: R Foundation for Statistical Computing).

S14. Bates, D., Maechler, M., and Bolker, B. (2013). lme4: Linear mixed-effects models using S4 classes. Retrieved from: http://CRAN.R-project.org/package=lme4 (R package version 0.999999-2).

S15. Nussey, D.H., Coulson, T., Festa-Bianchet, M., and Gaillard, J.M. (2008). Measuring senescence in wild animal populations: towards a longitudinal approach. Funct. Ecol. *22*, 393-406.

S16. Burgess, M.D., Nicoll, M.A.C., Jones, C.G., and Norris, K. (2008). Restricted dispersal reduces the strength of spatial density dependence in a tropical bird population. Proc. Biol. Sci. *275*, 1209-1216.

S17. Nevoux, M., Gimenez, O., Arlt, D., Nicoll, M.A.C., Jones, C., and Norris, K. (2011). Population regulation of territorial species: both site dependence and interference mechanisms matter. Proc. Biol. Sci. *278* 2173-2181.

S18. Nicoll, M.A.C. (2004). The ecology and management of a re-introduced population of the Mauritius kestrel (*Falco punctatus*), PhD Thesis, University of Reading.

S19. Senapathi, D., Nicoll, M.A.C., Teplitsky, C., Jones, C.G., and Norris, K. (2011). Climate change and the risks associated with delayed breeding in a tropical wild bird population. Proc. Biol. Sci. *278*, 3184-3190.

S20. Forslund, P., and Pärt, T. (1995). Age and reproduction in birds - hypotheses and tests. Trends Ecol. Evol. *10*, 374-378.

S21. Reid, J.M., Bignal, E.M., Bignal, S., McCracken, D.I., and Monaghan, P. (2003). Age-specific reproductive performance in red-billed choughs *Pyrrhocorax pyrrhocorax*: patterns and processes in a natural population. J Anim. Ecol. *72*, 765-776.

S22. Van de Pol, M., and Verhulst, S. (2006). Age-dependent traits: a new statistical model to separate within- and between-individual effects. Am. Nat. *167*, 766-773.

S23. Cam, E., Link, W.A., Cooch, E.G., Monnat, J.-Y., and Danchin, E. (2002). Individual covariation in life-history traits: seeing the trees despite the forest. Am. Nat. *159*, 96-105.

S24. Wood, S. (2013). Package 'mgcv': mixed GAM computation vehicle with GCV/AIC/REML smoothness estimation http://CRAN.R-project.org/package=mgcv (R package version 1.7-24).

S25. Bouwhuis, S., Sheldon, B.C., Verhulst, S., and Charmantier, A. (2009). Great tits growing old: selective disappearance and the partitioning of senescence to stages within the breeding cycle. Proc. Biol. Sci. *276*, 2769-2777.

S26. White, G.C., and Burnham, K.P. (1999). Program MARK: survival estimation from populations of marked animals. Bird Study *46*, 120-138.

S27. Cooch, E.G., and White, G. (2006). Program MARK – a 'Gentle Introduction', 4th edition. Available at: http://www.phidot.org/software/mark/docs/book/.
